# Psychosocial Problems in People Living with Thalassemia: A Systematic Review

**DOI:** 10.1177/23779608251323811

**Published:** 2025-03-19

**Authors:** Karolus Wangi, Rinanda Shaleha, Eri Wijaya, Barbara Birriel

**Affiliations:** 1 Ross and Carol Nese College of Nursing, Pennsylvania State University, University Park, PA, USA; 2 360168 Human Development and Family Studies, Pennsylvania State University, University Park, PA, USA; 3 Center for Epidemiology and Population Health Studies, Faculty of Public Health, Hasanuddin University, Makassar, Indonesia; 4 Ross and Carol Nese College of Nursing, Pennsylvania State University, Hershey, PA, USA

**Keywords:** thalassemia, patients, psychosocial problems, systematic literature review, psychological, burden

## Abstract

**Background:**

Thalassemia is a genetic disease that is inherited in families and has been designated as a global burden. Individuals living with thalassemia may experience impacts on their psychosocial well-being. However, there is a gap of limited study at the systematic review level regarding the extent to which psychosocial aspects contribute to the overall problems and burdens experienced by people living with thalassemia.

**Aim:**

This study aimed to synthesize the evidence on psychosocial problems in people living with thalassemia to provide comprehensive insight.

**Methods:**

The Preferred Reporting Items for Systematic Reviews and Meta-Analyses guideline was used to guide this systematic review. All relevant empirical studies in the English language that assessed the variety of psychosocial aspects were included. A total of 1,466 articles were identified from PubMed, CINAHL, ProQuest, PsycINFO, Scopus, Web of Science, and Cochrane databases from those inceptions to 6 March 2023. 1,443 articles were excluded due to duplication, not thalassemia- or psychosocial-related, availability of full-text, and not including patients. Finally, 13 studies were included and assessed using Mixed Methods Appraisal Tool (MMAT) appraisal tools. This study is already registered in International Prospective Register of Systematic Reviews (CRD42023431082).

**Results:**

This review analyzed 13 full-text studies conducted between 1993 and 2022. The MMAT quality assessment rated all 13 studies as high quality. The included studies used various designs, with six non-randomized quantitative studies, five descriptive quantitative studies, and two qualitative studies. The review summarizes each study's main findings, highlighting psychosocial problems and related outcomes, such as adherence and psychosocial morbidity, anxiety, educational and social impairment, coping style, internalizing and externalizing behaviors, and adaptive behaviors and family relationships.

**Discussion:**

The available literature globally highlights the psychosocial challenges of people living with thalassemia, particularly those who require regular blood transfusions across different timeframes. Despite inconsistent definitions across studies, it is notable that patients with thalassemia major facing physical changes associated with facial defects and experience significant psychosocial issues related to self-image, social interactions, and relationships that influence quality of life.

## Background

Thalassemia is a type of genetic disease that runs in families. The World Health Organization's (WHO) annual report in 2008 found that more than 40,000 babies each year are born with thalassemia, 25,500 of which are dependent on blood transfusions (Kattamis et al., 2020; Modell & Darlison, 2008). The WHO also estimated that in the worldwide population, at least 50 million people, across 60 countries, are thalassemia carriers (Ebrahim et al., 2019). Globally, WHO has highlighted thalassemia as a significant health problem in its assessment of the global burden of disease (Tuo et al., 2024).

Thalassemia involves routine blood transfusions, activity limitations, pain, fear of death, anxiety, hopelessness, and depression (Tarım & Öz, 2022). These conditions create emotional and psychological fear in patients, which requires medical and social support (Ul Hassan Rashid et al., 2020). Patients are often concerned about their lives and families, where they are trapped in a circle of discontent with an overwhelming expectation of death (Ahmadi et al., 2020).

Thalassemia causes considerable medical, financial, and psychosocial lifelong burdens related to the chronicity of the disease (Bansal, 2018; Tarım & Öz, 2022; Mevada et al., 2016). Psychosocial factors encompass elements that influence an individual's mental and social well-being, such as emotional resources and social support, which play essential roles in health behaviors (Thomas et al., 2020). Traditionally, “psychosocial” pertains to aspects of social adaptation and interpersonal relationships (Frosh, 2003). In this review, “psychosocial” is viewed as a dynamic interplay where mental and social dimensions reciprocally shape one another, a perspective that underscores the layered impacts of these factors on individuals with thalassemia.

Ironically, psychosocial problems in thalassemia are an iceberg phenomenon, where not all issues are visible or receive attention. This complexity may impact patients’ quality of life because they know their physical quality of life is decreasing gradually, although outcomes can vary significantly with optimal care. Moreover, psychosocial aspects are an essential part of quality of life in people with thalassemia, and more scholarly attention is needed in this area. Recently, several empirical studies on psychosocial issues have been conducted regarding people with this condition.

Nonetheless, based on our best knowledge and rapid review in several databases, there are few studies that have reviewed the psychosocial aspects in people with thalassemia in various contexts. These include (a) systematic review of psychosocial factors and chelation therapy adherence in thalassemia (Evangeli et al., 2010); (b) narrative review of psychosocial aspects in thalassemia patients and their quality of life (Alsaad, 2020); and (c) scoping review of psychosocial problems in adolescents with thalassemia major (TM) (Mardhiyah et al., 2023). There is still a gap between the three previous studies listed because there is no study that systematically reviews the literature regarding psychosocial problems in individuals living with thalassemia. To fill the gap, this study aimed to review the published studies regarding psychosocial problems in people living with thalassemia and present them systematically to gain comprehensive insight into psychosocial problems in this particular population.

## Methods

This systematic review referred to the Preferred Reporting Items for Systematic Reviews and Meta-Analyses guidelines (Supplementary) (Page et al., 2021). The protocol for this study was registered in the International Prospective Register of Systematic Reviews (PROSPERO) by the National Institute for Health Research with protocol registration number CRD42023431082. Record and updates are available from: https://www.crd.york.ac.uk/prospero/display_record.php?ID=CRD42023431082

### Information Source and Search Strategy

The literature review search was conducted by the lead author (K.W.) in March 2023. The search's date range began from the inception date of databases and went to 6 March 2023. In our search strategy, we used seven electronic databases. They were PubMed, CINAHL, ProQuest, PsycINFO, Scopus, Web of Science, and Cochrane. Two steps were applied and followed in the search strategy. Firstly, we involved a nursing librarian to brainstorm and discuss the keywords potentially used to build the search terms. The search terms were built on Boolean methods and divided into two terms. For the PubMed database, the search term was ((thalassemia OR thalaessemia OR thalassemia major OR beta thalassemia OR thalassemia dependent transfusion OR alpha thalassemia OR delta thalassemia) OR ("Thalassemia"[Mesh] OR "beta-Thalassemia"[Mesh] OR "delta-Thalassemia"[Mesh] OR "alpha-Thalassemia"[Mesh])) AND (((psychosocial) OR ("Psychosocial Functioning"[Mesh] OR "Psychosocial Support Systems"[Mesh])). For CINAHL, ProQuest, PsycINFO, Scopus, Web of Science, and Cochrane were (thalassemia OR beta thalassemia OR alpha thalassemia OR delta thalassemia OR thalassemia major OR thalassemia dependent transfusion) AND (psychosocial OR psychosocial outcomes OR psychosocial burden OR psychosocial factors).

### Inclusion and Exclusion Criteria

All relevant articles were included but selected by some inclusion criteria, such as (a) studies that targeted people with all types of hemoglobin mutation of thalassemia but not limited to either major or minor stage; (b) studies that claim to assess a variety of psychosocial aspects as stated on their titled and/or objectives; (c) empirical research; and (d) studies published in the English language. Exclusion criteria included: (a) studies that did not include patients as subjects; (b) unavailable full-text and abstract; (c) review articles but not limited to systematic and non-systematic, book section/chapter, theses, magazine articles, conference papers, opinions, abstracts, posters, letter, index, and proceedings.

### Study Screening and Selection

The articles searched from involved databases were imported, and the duplicates were removed through EndNote software. The two reviewers (K.W.) and (E.W.) independently read titles and abstracts and then reviewed the full-text as a filtered references process. If no consensus was reached, a third reviewer (R.S.) was invited and acted as an arbiter to resolve the discrepancies.

### Data Extraction

Data extracted included studies and reported as characteristic of the study that presents authors, country, study type, purpose of study, participants, thalassemia type, thalassemia stage, psychosocial aspect/variables measured, measurement tools, and relevant finding as shown in Table 1. Based on Table 1, two researchers (K.W.) and (R.S.) extracted, expanded, and compiled information to present in the Result section.

**Table 1.** Characteristic of Study.

Authors, year & country	Study type	Purpose of study	Participants, thalassemia type, and stage	Psychosocial aspect/variable(s) measured & measurement tool	Findings most relevant to this review
Al Kloub et al. (2014) Jordan	Quantitative non-randomized studies (Cross-sectional analytic study)	To explore the relationship between psychosocial status, disease knowledge, and adherence to deferoxamine treatment in adolescents with thalassemia major (TM)	**Participants:** 36 adolescents (22 females and 14 males); Age range 12-19 years old **Thalassemia type:** beta-thalassemia **Thalassemia stage:** major	**Aspect:** The psychosocial impairment **Tools:** PSC (Pediatric Symptoms Checklist)	**Psychosocial problems:** Psychosocial impairment problem: 36% of the adolescents had a PSC score greater than the recommended cut-off point Significant factors associated with psychological impairment: Adolescents > 16 years Low DFO adherence Mean ferritin > 2500 μg/L More than seven follow-up visits Family size more than six Family income of ≤ 350 JD Having siblings with thalassemia
Aydin et al. (1997) Turkey	Quantitative non-randomized studies (Case-control)	To assess the mental capacity, self-image, hopelessness, and anxiety displayed by children who suffered from TM, and to investigate the existence of psychiatric disorders in these children	**Participants:** ***Case****:* 25 children with TM Sex: 16 males and 9 females. Age: range 12-19 years old ***Control****:* 15 subjects matched. Sex: not identified. Age: 12-19 years old **Thalassemia type:** Not identified **Thalassemia stage:** major	**Aspect:** Self-image Hopelessness Anxiety General distress **Tools:** Offer Self-Image Questionnaire Beck Hopelessness Scale Trait Anxiety Inventory SCL-90-R Symptom Check List (revised)	**Psychosocial problems:** Self-image problem: Significantly lower in patients with TM than in control cases (p < 0.01) Hopelessness problem: Higher in patients with TM than in control cases (p <0.01) Anxiety problem: Higher in patients with TM than in control cases (p < 0.05) General distress problem: Higher in thalassemic patients, but not significant (P > 0.05)
Aydinok et al. (2005) Turkey	Quantitative descriptive studies (Survey)	To evaluate the psychosocial burden as well as to disclose whether the psychological status of the patients contributes to compliance with the therapy or to the contrary	**Participants:** 38 patients with TM (20 females & 18 males); Age range 6-18 years old and their mothers **Thalassemia type:** not specified **Thalassemia stage:** major	**Aspect:** Psychosocial burden: Anxiety-depression Aggression Internalizing **Tools:** Child Behavior Check-List (CBCL) SCL-90	**Psychosocial problems:** Deferoxamine (DFO) compliance: Anxiety-depression: p = 0.003 Aggression: p = 0.011 Internalizing: p = 0.009 Significantly higher scores were observed of DFO-good compliant patients compared with poor compliance.
Beratis (1993) Greek	Quantitative non-randomized studies (Case-control)	To examine the rate and nature of psychopathology in an unselected group of children with beta-thalassemia and assess their psychosocial functioning regarding relatedness and age-appropriate activities.	**Participants:** ***Case:*** 57 children with TM. Sex: 29 males and 28 females. Age: range 5-12 years old ***Control:*** 57 subjects matched. Sex: not identified. Age: 5-12 years old **Thalassemia type:** beta-thalassemia **Thalassemia stage:** not specified	**Aspect:** Psychiatric disorders **Tools:** Semi-structured interview using Diagnostic and Statistical Manual III-R (DSM-III-R)	**Psychosocial problems:** ➢ Activities Behavior: Sports child likes: p = 0.17 Sports participation skills: p = 0.11 Non-sports activities: p = 0.44 Activities participation skills: p = 0.38 ➢ Social Behavior Number of friends p = 0.26 Contacts with friends p = 0.41 Behavior with others p = 0.0005 Play and work p = 0.44 Thalassemic group: ➢ Disorder Oppositional Defiant Disorder (ODD): 13 patients Separation Anxiety Disorder (SAD): 8 patients Primary Functional Enuresis (PFE): 7 patients Attention Deficit Hyperactivity Disorder (ADHD): 2 patients Sleep Terror Disorder (STD): 1 patient Stuttering (St): - Tension Headache (TH): - Control group: Oppositional Defiant Disorder (ODD): 3 patients Separation Anxiety Disorder (SAD): 5 patients Primary Functional Enuresis (PFE): 5 patients Attention Deficit Hyperactivity Disorder (ADHD): 0 patient Sleep Terror Disorder (STD): 0 patient Stuttering (St): 1 patient Tension Headache (TH): 1 patient
Canatan et al. (2003) Turkey	Quantitative descriptive studies	To analyze the psychosocial burden of thalassemia major from a developing country’s perspective in a major thalassemia center in Antalya, south Turkey	**Participants:** 99 children (1-15 years old) 32 adults (16-27 years old) **Thalassemia type:** beta-thalassemia **Thalassemia stage:** Major	**Aspect:** psychosocial burden **Tools:** Preexisting questionnaire. Three versions are available: (A) a parent report form for patients under 16 years of age; (B) a self-report form for adult patients; and (C) a form for parents to report their own situation	**Psychosocial problems:** Children: Education: 60% Time of school: 64% Sport: 29% Anxiety: 31% Family interaction: 3% Social isolation: 12% Social life/interaction: 20% Feelings of difference: 24% Stigmatization: 2% Social integration: - Self-image: - Denial: - Confusion: - Feelings of guilt: - Finances: - Family size: - Prenatal diagnosis: - Adults: Education: 47% Time of school: 47% Sport: 69% Anxiety: 84% Family interaction: 6% Social isolation: 19% Social life/interaction: 25% Feelings of difference: 50% Stigmatization: 19% Social integration: 47% Self-image: 67% Denial: 25% Confusion: - Feelings of guilt: - Finances: - Family size: - Prenatal diagnosis: -
Cerami et al. (2022) Italy	Quantitative descriptive studies (case-control)	To explore distress, anxiety, depression, loneliness, coping strategies, and changes in life habits of thalassemia patients during Covid-19 pandemic	**Participants:** ***Case:*** 43 patients. Sex: 9 males & 3 females. Age range 18-55 years old ***Control:*** 86 healthy subjects. Sex: 18 males & 64 females. Age range 20-57 years old **Thalassemia type:** beta-thalassemia **Thalassemia stage:** major	**Aspect:** Psychosocial burden Global distress Loneliness Coping styles **Tools:** Online questionnaire Depression Anxiety Stress Scales-21 (DASS-21) Italian Loneliness Scale (ILS) Italian version of the Coping Orientation to the Problems Experienced (COPE-NVI-25)	**Psychosocial problems:** a. Global distress Anxiety: p = 0.013 Distress: p = 0.302 Depression: p = 0.079 b. Loneliness Social loneliness: p = 0.800 Emotional loneliness: p = 0.947 General loneliness: p = 0.397 c. Coping styles Positive attitude: p = 0.831 Social support: p = 0.196 Problem orientation: p = 0.078 Transcendences orientation: p < 0.001 Avoidance strategies: p = 0.142
Di Palma et al. (1998) Italy	Qualitative studies (descriptive)	To explore the effect of thalassemia major on the psychosocial adjustment of adolescents and young adults	**Participants:** **Group A:** ***Case:*** 90 unmarried patients. Sex: 45 males & 45 females. Age range 14–22 years old ***Control:*** 100 matched subjects **Group B:** ***Case:*** 19 thalassemic married subjects. Sex: 6 males & 13 females. Age range 28–45 years old of which 7 patients had children, and 12 did not. **Thalassemia type:** beta-thalassemia **Thalassemia stage:** major	**Aspect:** psychosocial adjustment **Tools:** Semi-structured interviews using *ad hoc* questionnaire	**Psychosocial problems:** **Group A:** Social adjustment The case had normal psychological and social development and scored better than control Self-esteem The case had normal psychological and social development and scored better than control Self-description The case had normal psychological and social development and scored better than control Family relationships The case group is stronger than normal controls **Group B:** The behavior of thalassemic couples did not differ from that observed in non-thalassemic couples in previous investigations
Elzaree et al. (2018) Egypt	Quantitative non-randomized studies (Case-control study)	To assess the psychosocial burden and the adaptive functioning in children with beta-thalassemia major	**Participants:** **Case:** 50 children. Sex: 25 males and 25 females. Age range 5–17 years old ***Control:*** 50 normal children **Thalassemia type:** beta-thalassemia **Thalassemia stage:** major	**Aspect:** Adaptive functions. Psychosocial morbidity. **Tools:** Vineland Adaptive Functioning Scale Pediatric Symptom Checklist (PSCL)	**Psychosocial problems:** Total adaptive behavior: p = 0.008 PSCL between groups p = 0.06
Khurana et al. (2006) India	Qualitative studies (descriptive)	To study the psychosocial life aspects of Indian adolescents suffering from transfusion-dependent beta-thalassemia major	**Participants:** 50 adolescents. Sex: 25 males and 25 females. Age range 12–20 years old **Thalassemia type:** beta-thalassemia **Thalassemia stage:** major	**Aspect:** psychosocial burden **Tools:** Structured interviews	**Psychosocial problems:** Education: 70% had an adverse impact on education Sport activities: over 2/3 'a related physical weakness to their disease Body image: 68% negative self-concept Family interactions: - Social life: 80% reported no impact on social life Disease and complications 100% worried about life-threatening complications 56% concerned about future health 34% concerned about education Coping Source of strength in parents and God 82% did not discuss their illness with friends Financial and medical support: 20% affected, denied benefits of optimal medical management
Messina et al. (2008) Italy	Quantitative descriptive studies (Survey)	To assess the self-image, the quality of life, the way of coping and to investigate the existence of psychiatric disorders in young adults with thalassemia major	**Participants:** 147 young adult patients. Sex: 57 males and 90 females. Age range 18–73 years old **Thalassemia type:** beta-thalassemia **Thalassemia stage:** major	**Aspect:** psychosocial burden Psychiatric disorders The way of coping Self-image Quality of life **Tools:** Symptom check list-90 revised (SCL-90-R) Coping questionnaire (WCQ) The Machover's test The short form 36-health survey questionnaire (SF-36)	**Psychosocial problems:** Psychiatric disorders (mean value) Somatization-SOM (0.7) Obsessive-compulsive disorder-DOC (0.74) Interpersonal sensitivity-IS (0.56) Depression-DEP (0.72) Anxiety-ANX (0.6) Paranoid ideation-PAR (0.71) Hostility-HOS (0.52) Fobica anxiety-PHOB (0.72) Psychoticism-PSY (0.34) Global severity index-GSI (0.60) Positive syndrome distress index-PSDI (1.55) Positive symptom total-PST (32.16) The way of coping (percent value) Escape-avoidance (43%) Self-controlling (26%) Seeking Social Support (12%) Distancing (11%) Confrontive Coping (8%) Accepting Responsibility (0%) Painful Problem Solving (0%) Positive Reappraisal (0%) Self-image (the Machover's test) Difficulties of social relationships and tendency to keep on. Feeling of precarity and fragility Quality of life (mean value) Physical functioning (78.92) Role limitations due to physical problems (68.75) Body pain (83.03) General health (65.47) Vitality (52.85) Social functioning (37.32) Role limitations due to emotional problems (35.89) Mental health (66.71)
Raman et al. (2019) India	Quantitative non-randomized studies (Case-control study)	To detect psychosocial issues that prompt referral for counseling	**Participants:** ***Case:*** 30 children. Sex: 16 males and 14 females. Age range 5–18 years old ***Control:*** 30 subjects matched age and sex **Thalassemia type:** Not specified **Thalassemia stage:** major	**Aspect:** Psychosocial morbidity **Tools:** The Pediatric Symptom Checklist (PSC-17) The Strengths and Difficulties Questionnaire (SDQ) The General Health Questionnaire (GHQ)	**Psychosocial problems:** PSC PSC total (case: 9.93 ± 4.83, control: 4.87 ± 3.74, P < 0.001) PSD internalizing (case: 2.5 ± 1.98, control: 1.23 ± 1.17, p = 0.004) PSC externalizing (case: 3.70 ± 2.95, control: 1.57 ± 1.98, P = 0.002) PSC attention (case: 3.73 ± 2.13, control: 2.03 ± 1.54, P = 0.001) SDQ SDQ total (case: 14.9 ± 6.75, control: 10.9 ± 4.2, P = 0.008) Emotional problem score (case: 3.97 ± 2.75, control: 2.2 ± 1.83, P = 0.05) Conduct problem score (case: 3.73 ± 1.89, control: 2.67 ± 1.37, P = 0.015) Hyperactivity score (case: 4.73 ± 2.10, control: 4.6 ± 2.42, P = 0.820) Peership problem score (case: 2.5 ± 1.93, 1.43 ± control: 1.36, P = 0.016) GHQ-12 (case: 10.3 ± 4.7, control: 8.0 ± 3.0, P = 0.027)
Ratip et al. (1995) UK	Quantitative descriptive studies (Survey)	To evaluate psychosocial burden among patients with thalassemia intermedia and their parents	**Participants:** 28 patients. Sex: 12 males and 16 females. Age range 5–66 years old **Thalassemia type:** beta-thalassemia **Thalassemia stage:** intermedia	**Aspect:** Psychosocial burden of adult patients **Tools:** specifically designed questionnaires	**Psychosocial problems:** Adult patients: Unaffected (48%) Education (43%) Sports activity (62%) Stigmatization (67%) Anxiety (57%) Reduce social activities (28%)
Saini et al. (2007) India	Quantitative non-randomized studies (Case-control study)	To assess the prevalence and the spectrum of psychosocial morbidity and its correlation with various social and disease-related factors in children with beta-thalassemia major	**Participants:** ***Case:*** 60 children with transfusion-dependent β-thalassemia. Age range 5–15 years old ***Control:*** 60 children of matched age group and social background **Thalassemia type:** beta-thalassemia **Thalassemia stage:** major	**Aspect:** Psychosocial morbidity **Tools:** Semi-structured interview Pediatric Symptom Checklist (PSC) Childhood Psychopathology Measurement Schedule (CPMS)	**Psychosocial problems:** PSC (P < .001) (mean value) Case: 11.63 ± 3.79 (range, 7–24) Control: 5.78 ± 2.572 (range, 2–13) CPMS (P < .001) (mean value) Case: 11.63 ± 3.6 (range, 6–25) 54% had a mean score ≥10 Control: 6.08 ± 2.8 (range, 1–14) 8.3% had a mean score ≥10

### Quality Assessment for Literature

In this study, we used the Mixed Methods Appraisal Tool (MMAT) for quality assessment of all the included studies (Hong et al., 2018). This appraisal tool consists of two screening questions for all types of study and five quality assessment questions in each of the five categories of studies, including qualitative, quantitative randomized controlled trials, quantitative non-randomized, quantitative descriptive, and mixed-methods studies (Hong et al., 2018). The review process starts by using the screening questions to review the suitable study that is potentially assessed by MMAT tools. Then, all the reviewed studies were assessed using the five quality assessment questions based on the appropriate category for each study. The two reviewers (R.S.) and (E.W.) independently assessed the quality of the studies, including the methodology used. Three types of ratings are used as criteria. Those include “yes,” “no,” or “cannot tell.” The discrepancies found were reconciled via discussion and consensus. To summarize the total score of each study, the approach from Pluye et al. (2009) was used. The criteria rated “yes,” scored as 1. The criteria rated “no” is equal to “cannot tell” (Hong et al., 2018), and these scored as 0. The simple calculation was used by dividing the score answered with the quality assessment questions (score/7) and presented as a percentage (%). In terms of categorizing the total percentage score, there was no specific interpretation of scoring, so the three levels to categorize the scoring system by low (<35%), medium (36–70%), high (71–100%) were used (D'Amen et al., 2021).

## Result

### Selection of the Study

**Figure 1.**
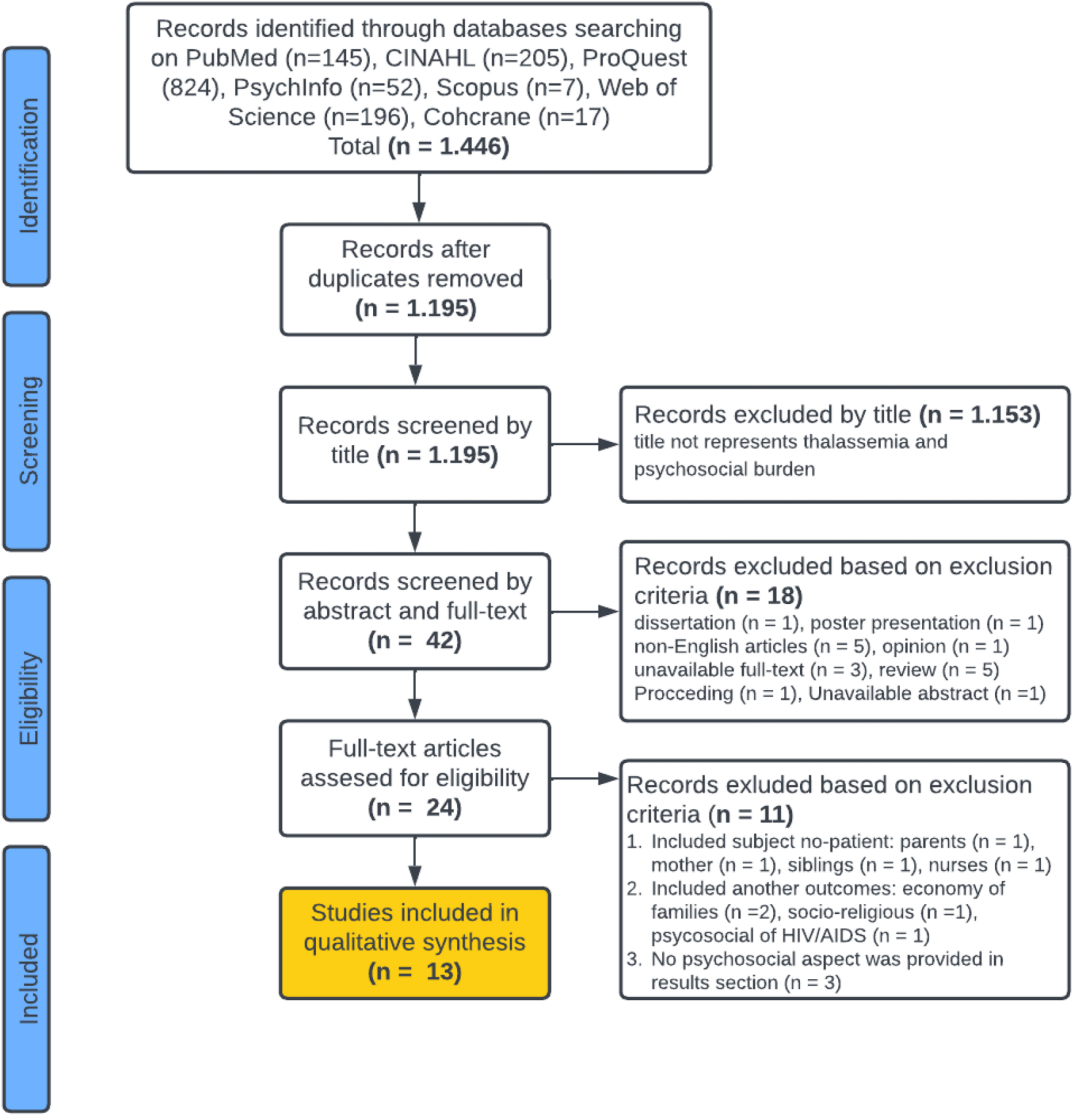
Flowchart of identified and selected studies.

The database search resulted in 1,446 records and became 1,195 after removing the duplicate files. Following the title screening, 1,153 records were eliminated because they did not represent thalassemia or psychological burden/problems. After an abstract and full-text screening process, we excluded 18 papers consisting of a dissertation (n = 1), a presentation poster (n = 1), a non-English article (n = 5), an opinion (n = 1), unavailable full-text (n = 3), reviews (n = 5), a proceeding (n = 1), and unavailable abstract (n = 1). The remaining 42 become 24 records. Of these 24 records, we removed 11 papers because there were articles involving non-patient subjects, such as the parent (n = 1), mother (n = 1), sibling (n = 1), and nurse (n = 1). Moreover, four articles included other outcomes such as economy of families (n = 2), socio-religious (n = 1), and psychosocial of HIV/AIDS (n = 1), and the last three articles have no discussion of psychosocial aspects in their result section (n = 3). Finally, the qualitative synthesis included 13 records. The literature search and screening process is depicted in Figure 1.

### Characteristics of the Included Studies and Quality Appraisals

This systematic review synthesized evidence from 13 studies on people with thalassemia that were published from 1993 (Beratis, 1993) to 2022 (Cerami et al., 2022). Six of these papers are non-randomized quantitative studies (Al-Kloub et al., 2014; Aydin et al., 1997; Beratis, 1993; Elzaree et al., 2018; Raman et al., 2019; Saini et al., 2007), five papers are descriptive quantitative studies (Aydinok et al., 2005; Canatan et al., 2003; Cerami et al., 2022; Messina et al., 2008; Ratip et al., 1995), and two papers are qualitative studies (Di Palma et al., 1998; Khurana et al., 2006). In Table 1, we summarize the essential features and key findings of the selected study, including the psychosocial problems each study measured and how they were described.

### Quality Assessment

To evaluate the quality of the included studies, we used the MMAT (Hong et al., 2018). The five core quality criteria are divided into five categories of study designs with a methodological focus and present two pre-screening questions for each. The 13 articles included are considered high quality (Table 2). Furthermore, all the data define research questions clearly and address their research questions. The sample represents the population and uses appropriate measurements.

**Table 2.** Quality Assessment Articles.

Articles	Screening 1	Screening 2	Criteria 1	Criteria 2	Criteria 3	Criteria 4	Criteria 5	Total score	Category
	Clarity of research question	Data address research question	Relevance of sampling strategy to addressing research question	Representation of sample to the population	Appropriateness of measurements	Low risk of non-responsive bias	Appropriateness of statistical analysis		
Al-Kloub et al., 2014	Yes	Yes	Yes	Yes	Yes	Yes	Yes	100%	High
Aydin et al., 1997	Yes	Yes	No	Yes	Yes	Yes	No	71.4%	High
Aydinok et al., 2005	Yes	Yes	Yes	Yes	Yes	No	Yes	85.7%	High
Beratis, 1993	Yes	Yes	Yes	Yes	Yes	Yes	Yes	100%	High
Canatan et al., 2003	Yes	Yes	Yes	Yes	Yes	Yes	Yes	85.7%	High
Cerami et al., 2022	Yes	Yes	Yes	Yes	Yes	No	Yes	85.7%	High
Di Palma et al., 1998	Yes	Yes	Yes	Yes	Yes	Yes	Yes	100%	High
Elzaree et al., 2018	Yes	Yes	Yes	Yes	Yes	Yes	Yes	100%	High
Khurana et al., 2006	Yes	Yes	Yes	Yes	Yes	Yes	Yes	85.7%	High
Messina et al., 2008	Yes	Yes	Yes	Yes	Yes	No	Yes	85.7%	High
Raman et al., 2019	Yes	Yes	Yes	Yes	Yes	Yes	Yes	100%	High
Ratip et al., 1995	Yes	Yes	Yes	Yes	Yes	Yes	Yes	100%	High
Saini et al., 2007	Yes	Yes	Yes	Yes	Yes	Yes	Yes	100%	High

### Type of Thalassemia and Association with Psychosocial Problems

All studies have included the types of thalassemia. Eight studies (Al-Kloub et al., 2014; Canatan et al., 2003; Cerami et al., 2022; Di Palma et al., 1998; Elzaree et al., 2018; Khurana et al., 2006; Messina et al., 2008; Saini et al., 2007) involved participants with type thalassemia beta stage major. However, the rest of the other studies were incomplete in mentioning the type of thalassemia studied therein. Aydin et al. (1997), Raman et al. (2019), and Aydinok et al. (2005) only mention the stage, which is major, but they do not identify the type of thalassemia. Then, Beratis (1993) describes the type of thalassemia, which is beta-thalassemia, but the stage is not specified. Lastly, the type of thalassemia in Ratip et al. (1995) is beta-thalassemia and stage intermedia. Additionally, several studies also involved the participants’ parents (Aydinok et al., 2005; Canatan et al., 2003; Ratip et al., 1995). Most studies included individuals with TM because, at this stage, patients typically have severe anemia and hemoglobin levels below 9 mg/dl, necessitating an immediate blood transfusion.

### Psychosocial Morbidity and Adherence

Individuals with PSC (Pediatric Symptom Checklist) scores above the cut-off (>28) exhibited significantly higher psychosocial morbidity, independent of gender, and this morbidity is anticipated to rise with age, social factors, and disease-related factors, as evidenced by a positive correlation with the duration of illness, regular blood transfusion, and deferoxamine (DFO) transfusion. The idea of psychosocial morbidity emphasizes the importance of considering both psychological and social factors when evaluating and addressing a person's general health and well-being. It aligns with the findings of Saini et al. (2007) and Raman et al. (2019). Al-Kloub et al. (2014) found that most adolescents who are aware of their thalassemia are more likely to adhere to treatment. A study by Aydinok et al. (2005) also showed that adherence to treatment and high knowledge about the disease have psychosocial implications for patients with thalassemia (children and adolescents) and their mothers. Both patients (N = 38) and their mothers are reported to have high scores for depression, anxiety, aggression, internalizing problems, and the total problem of DFO-compliant patients compared with non-compliant as measured by Child Behavior Checklist scores, specifically DFO-compliant patients demonstrated higher scores in anxiety-depression (58.4 ± 41.5 vs. 54.3 ± 20.1, p < 0.05), aggression (51.9 ± 39.9 vs. 50.3 ± 19.6, p < 0.05), internalizing problems (53.1 ± 28.7 vs. 48.6 ± 17.7, p < 0.05), and total problem (48.6 ± 29.8 vs. 41.4 ± 23.4, p < 0.05). Furthermore, patients with thalassemia and their mothers have a higher prevalence of psychopathology than the general population, as indicated by the SCL-90R (Symptoms Checklist) score (0.73 ± 0.40 vs 0.43 ± 0.25, p < 0.05).

### Anxiety

In a study by Canatan et al. (2003), pediatric patients also experienced anxiety (31%), a sense of being out of normal (24%), limited participation in sports (29%), and were negatively affected in their social interactions (20%). Most of the adult patients (84%) also reported experiencing anxiety and having a feeling of difference from other people (50%). This result is in line with the study by Aydin et al. (1997) which reported that the participants also had higher anxiety compared to the control group (42.04 ± 5.45 vs. 37.53 ± 5.62 p < 0.05), which was evidence of psychosocial dysfunction. In addition, adults with thalassemia felt that their integration and social activities were disrupted. Some also experienced a denial phase. Additionally, the parents of the patient also experienced heightened anxiety. Approximately 82% reported experiencing anxiety, 47% felt their work was disrupted, 14% felt confused about their conditions, 6% experienced severe family adjustment, 26% experienced social isolation, and 1.8% experienced a marital breakdown. These psychosocial burdens experienced by parents were positively correlated with the psychosocial burdens handled by patients, and compared to mothers from middle-class families, mothers from low-income families reported higher anxiety levels (Aydinok et al., 2005).

### Education and Social Impairment

Canatan et al. (2003) found that the psychosocial burden was experienced by both, patients with thalassemia (99 children, 32 adults) and their parents (112 parents). This study found that most children of school age (60%) and adults (47%) were impacted in their education, primarily because they had to visit the hospital for regular blood transfusions. Another study found that children with thalassemia who performed poorly in school also had significantly higher levels of psychosocial maladjustment (Saini et al., 2007). The psychosocial effects on aspects of education and sports were also found in the study by Ratip et al. (1995). The study reported that around 43% of patients experienced educational disruptions, primarily due to the need to take time off from school. Slightly over half (57%) had to take time off school because of their illness. The duration of absences varied, ranging from 1 day or fewer to more than 1 week per month. Most of the affected patients expressed displeasure with their academic performance, feeling that they had fallen short of their potential, and they blamed their thalassemia condition for this relative failure. They also complained of tiredness and suboptimal performance (62% are affected) due to thalassemia.

### Coping Styles

Messina et al. (2008) show various coping styles for patients with thalassemia: escape-avoidance (43%), self-controlling (26%), seeking social support (12%), distancing (11%), and confrontive coping (8%). Moreover, the results of the SCL-90-R test show that psychopathological characteristics are present on various scales. These include somatization (SOM), obsessive-compulsive disorder (DOC), and depression (DEP). In addition, patients with thalassemia also experience difficulties in their social functioning and role limitations due to emotional problems. A study by Cerami et al. (2022), which measured psychosocial burden, shows that apart from anxiety and transcendence orientation, no significant differences were found in general distress (F[1,128] = 1.073, p = 0.302), depression (F[1,128] = 3.138, p = 0.079), general loneliness (F[1,128] = 0.723, p = 0.397), coping styles in the form of positive attitude (F[1,128] = 0.046, p = 0.831), social support (F[1,128] = 1.689, p = 0.196), problem orientation (F[1,128] = 3.158, p = 0.078), and avoidance strategies (F[1,128] = 2.188, p = 0.142). The patients need adaptive coping mechanisms. According to Lazarus (1993) transactional model of stress and coping, individual and environmental factors affect how situations are perceived and assessed. Stress appraisal and coping mechanisms are influenced by factors in the individual and the environment (such as beliefs, demands, and constraints), that impact physiological and emotional responses.

### Internalizing and Externalizing Behavior

Furthermore, research by Raman et al. (2019) found the internalizing (2.5 ± 1.98 vs. 1.23 ± 1.17, p < 0.05) and externalizing scores (3.70 ± 2.95 vs. 1.57 ± 1.98, p < 0.05), and were all higher in the study group than in the control group. Internalizing disorders are directed inward and are a sign of a child's psychological and emotional state, as opposed to externalizing behaviors, which are outwardly directed and reflect behavior toward the physical environment (Eisenberg et al., 2001; Liu et al., 2011). When examining the components of the SDQ (Strengths and Difficulties Questionnaire), it was found that children with TM had noticeably higher scores concerning emotional problems (3.73 ± 1.89 vs. 2.67 ± 1.37, p < 0.05), conduct problems (3.73 ± 1.89 vs. 2.67 ± 1.37, p < 0.05), and peer relationship problems (2.5 ± 1.93 vs. 1.43 ± 1.36, p < 0.05) compared to the control group. In addition, the parents of children with thalassemia scored higher on the General Health Questionnaire (GHQ) than the control group (10.3 ± 4.7 vs. 8.0 ± 3.0, p < 0.05).

### Adaptive Behavior and Family Relationship

A qualitative study by Elzaree et al. (2018) found that communication skills were the most impaired, with 38% of sick children scoring below average (70–84, mean = 85–115). Although no theoretical model or concept was used in their study to explain the variables they used, the conditions and characteristics of the study they conducted led them to conclude that adaptive function is a component of psychosocial variables. Another qualitative study by Di Palma et al. (1998) showed that the case group had normal psychological and social development and scored better than the control group. Psychological and social development are social adjustment, self-esteem, and self-description. Interestingly, family relationships in patients with thalassemia are stronger than those reported by normal control. Moreover, significant factors such as a family size of more than six (41% [10], p < 0.05), low family income which ≤350 JD (43% [12], p < 0.001), and siblings with thalassemia ≥2 (43% [9], p < 0.05) are associated with psychological impairment (Al-Kloub et al., 2014).

## Discussion

Psychosocial factors are characteristics that affect an individual psychologically and/or socially (Thomas et al., 2020). Psychosocial factors such as psychological resources and social support are crucial to understanding health behaviors. In conventional articles, the term “psychosocial” refers to addressing social adjustment or interpersonal relationships (Frosh, 2003). Based on that definition, “psychosocial” is the dynamic relationship between a person's psychological and social dimensions, where one influences the other. Moreover, Frosh (2003) argues that explorations of the psychosocial as a cohesive entity, in which traditionally separate concepts like "individual" and "society" are considered connected or even potentially indistinguishable, are relatively rare in psychological literature. This gap may be caused by the difficulty in conceptualizing the "psychosocial" as an intertwined phenomenon. There may be some incommensurability between the social and psychological domains that prevents complete integration. Additionally, because of the inherent complexity that each part of this combined entity possesses, it is possible that grappling with elusive concepts will result from letting go of the need to only work within the parameters of a disciplinary construct (such as the "individual" or "society").

Furthermore, psychosocial factors include protective and risk factors (Thomas et al., 2020). Social networks and social support are two examples of psychosocial resources in the social environment. Coping ability, mastery, sense of coherence, and self-esteem are important psychological resources. Furthermore, psychological risk factors include exhaustion, depression, hopelessness, and hostility. Based on that conceptual definition regarding psychosocial factors, psychosocial problems might be defined as the combination of the impact of psychological and social factors on an individual's well-being and functioning. It includes the emotional, cognitive, and social difficulties arising from a specific condition, event, or circumstance (such as, an individual with thalassemia).

Overall, from the 13 articles included in this review, there were some gaps in terms of the years between one study and another, which highlighted how quality of care works in different time frames, countries, and settings. However, on another level, the invasive supportive treatments and frequent exposure to care is related to the psychosocial condition of individuals living with thalassemia, particularly those who depend on blood transfusions to survive their entire life (Taher et al., 2021). This evidence highlights the importance of quality of long-term care for people with thalassemia. Additionally, those individuals with thalassemia who depend on blood transfusions, face various psychosocial challenges because of the uncertainty of the disease, which directly impacts their quality of life (Ahmadi et al., 2020). Consequently, they require social and moral support from family, medical personnel, and members of the community including all parties involved (Ul Hassan Rashid et al., 2020).

Demographically, the psychosocial condition of people living with thalassemia is also influenced by regional and socioeconomic factors, particularly in South Asia, where thalassemia prevalence is high. For instance, in India, patients face significant challenges due to limited access to safe and sufficient blood transfusions. As Datta and Sinha (2024) highlight, the lack of a national blood policy, insufficient alloantibody detection, and fragmented transfusion services contribute to under-transfusion and a higher risk of transfusion-transmitted infections. These systemic issues place an additional psychosocial burden on patients and their families, who often experience increased anxiety, financial strain, and social isolation due to frequent travel and healthcare costs. Addressing these unique circumstances is essential for developing targeted psychosocial interventions that can better meet the needs of thalassemia patients in resource-limited settings.

Additionally, Sarwer and Polonsky's (2016) study included psychosocial characteristics as a wide range of factors, such as signs of anxiety and depression, quality of life regarding one's health and weight, self-esteem, body image, and sexual function. These elements play a part in a person's overall well-being and sense of self because they show how psychological and social factors interact intricately. Most of the studies in this review addressed psychosocial variables. However, there needs to be a clear conceptual definition of which boundaries were included in psychosocial variables, which none of the 13 articles in this review provided. The boundaries within the scope are unclear because each article addresses various issues.

Suppose we use the broad definition of psychosocial as: the dynamic relationship between a person's psychological and social dimensions, where one influences the other, for all existing articles. In that case, psychosocial characteristics are included in all these articles. However, psychosocial significance in healthcare emphasizes the need for a clear and comprehensive definition to improve patient care and outcomes. According to the scholarly evidence, several authors stated that, it is challenging to conceptualize the term psychosocial because it appears to be an "intertwined entity, with all the imponderables it raises" (Frosh, 2003, p. 3). One of the reasons the term psychosocial is not clearly defined is that it is difficult to conceptualize a single definition. Moreover, Peter et al. (2022) explained that many attributes are associated with psychosocial health, including emotional well-being, social support, coping mechanisms, self-esteem, resilience, and cultural influences. Therefore, these things make it is more complex to establish clear psychosocial boundaries.

## Limitations and Strengths

This study had a limitation that there was no time restriction for the article search. This resulted in some articles included in the review that are more than 10 years old which might impact to the lack of discussion related to quality of care which influenced by different time frames, countries, and settings. At the same time, this limitation could be reflected as a strength of this review because all the articles from its inception in selected electronic databases were included, representing all the recorded psychosocial studies in people with thalassemia worldwide on those data bases. Another strength is, this is the first literature systematic study that review the psychosocial problems in people with thalassemia, to the best of our knowledge.

## Conclusion

The results of this literature review found that 13 studies related to the psychosocial problems of people with thalassemia have been conducted. We conclude that several psychosocial characteristics are reflected in the studies, but the conceptual definition of the psychosocial term was not clearly defined. For future directions, consider our finding that the psychosocial term was not clearly stated and well-defined in each study included: we suggest conducting a conceptual review of the psychosocial term, particularly in people with thalassemia to arrive at a standard definition. Also, when anticipating further psychosocial study while the conceptual definition of the psychosocial term is still missing, building a “operational definition” in the study plan might be an alternative.

## Supplemental Material

sj-docx-1-son-10.1177_23779608251323811 - Supplemental material for Psychosocial Problems in People Living with Thalassemia: A Systematic Review Supplemental material, sj-docx-1-son-10.1177_23779608251323811 for Psychosocial Problems in People Living with Thalassemia: A Systematic Review by Karolus Wangi, Rinanda Shaleha, Eri Wijaya and Barbara Birriel in SAGE Open Nursing
